# Lycium barbarum polysaccharide protects HSF cells against ultraviolet-induced damage through the activation of Nrf2

**DOI:** 10.1186/s11658-018-0084-2

**Published:** 2018-05-01

**Authors:** Bihua Liang, Liqian Peng, Runxiang Li, Huaping Li, Ziyin Mo, Xinyue Dai, Na Jiang, Qing Liu, Erting Zhang, Huiyan Deng, Zhenjie Li, Huilan Zhu

**Affiliations:** 1 Guangzhou Institute of Dermatology, Guangzhou, China; 2 Guangzhou Medical University, Guangzhou, China; 3 Guangzhou First People’s Hospital, Guangzhou, China; 4 The Fifth Affiliated Hospital of Guangzhou Medical University, Guangzhou, China; 5 The Third Affiliated Hospital of Sun Yat-sen University, Guangzhou, China

**Keywords:** Ultraviolet damage, *Lycium barbarum*, Polysaccharide, Nrf2

## Abstract

**Background:**

*Lycium barbarum* polysaccharide (LBP) is considered an antioxidant agent. NF-E2-related factor-2 (Nrf2) is an important regulator for protection against UV damage. In this study, we verified the performance of LBP and the correlation between LBP and Nrf2.

**Methods:**

HSF cells were treated with LBP to determine dose and time dependencies. An antioxidant response element (ARE) reporter was designed to monitor the activity of the Nrf2 antioxidant pathway.

**Results:**

For HSF cells, the optimal LBP treatment was 300 μg/ml for 3 h. The ARE-reporter assay showed that LBP could increase the robustness of p-Nrf2. Treatments with genistein and LY294002 reduced of nuclear p-Nrf2 after 24 h. LBP increased the level of nuclear Nrf2, which functions by both phosphorylation and nuclear translocation. Silencing Nrf2 led to increased reactive oxygen species (ROS) levels, lower cell viability, and decreased superoxide dismutase (SOD) and glutathione peroxidase (GSP-PX) levels. This induced a higher level of lipid peroxide (LPO). However, LBP could decrease the levels of ROS and LPO and enhance the levels of SOD and GSP-PX.

**Conclusion:**

LBP protects HSF cells against UV damage via the regulation of Nrf2.

## Background

The skin is continuously exposed to environmental biotic and abiotic factors, but it has developed protective mechanisms to cope with these [1]. Ultraviolet radiation (UVR) is a prominent environmental agent. It continually affects cellular homeostasis in human skin [2]. The radiation that reaches the earth’s surface is mostly UVA and UVB. UVA is normally in the wavelength range from 320 to 400 nm and causes skin tanning effects. UVB is in the range from 280 to 320 nm and causes sunburn (erythema) [3]. UVA is known to damage DNA, protein and lipids. Excessive UVB exposure can cause skin cancers, basal cell carcinoma and squamous cell carcinoma [4]. UVR also stimulates cortisol production in human skin keratinocytes and melanocytes with predictable involvement in local carcinogenesis [5]. Nuclear factor erythroid 2-related factor 2 (Nrf2) plays an important role in cellular defense against various harmful stimuli, including radiation [6]. As a transcription factor, it is generally located in the cytoplasm and binds to its inhibitor, Kelch-like ECH-associated protein 1 (Keap1). Keap1 is related to the actin cytoskeleton and functions to prevents Nrf2 entry into the nucleus. However, under conditions of stress, Nrf2 escapes from Keap1 and translocates into the nucleus, where it binds to the antioxidant-response element (ARE) and upregulates the transcription of protective factors [7, 8]. Recent studies have confirmed that the protective effect against UVR-induced damage relies on the functions of the Nrf2-dependent pathway, indicating its importance in the protective process [9, 10].

*Lycium barbarum* polysaccharide (LBP) is the main bioactive component of *L. barbarum*. It is composed of arabinose, glucose, galactose, mannose, xylose and rhamnose [11]. It has multiple biological and pharmacological effects, including anticancer [12–14], antifatigue [15], neuroprotective [16, 17], antioxidant [18, 19], hypoglycemic [20, 21], fertility-protective [22] and immunomodulating [23] functions. Recent studies have indicated that LBP has protective effects against ionizing radiation-induced damage. For example, it was shown to protect reproductive function, prevent radiation-induced spermatogenic cell apoptosis, and enhance self-repair of the testis in vivo [24]. The mechanism underlying LBP’s protective effect against radiation remains unclear.

We hypothesized that the protective effect of LBP against UVR-induced damage is related to the functions of Nrf2. Therefore, we designed assays using the highly fibroblastic HSF cell line to evaluate Nrf2 expression before and after LBP treatment. Gene silencing of Nrf2 was also conducted. Physiological features that were measured included cell viability and the levels of superoxide dismutase (SOD), reactive oxygen species (ROS), glutathione peroxidase and lipid peroxide. The aim was to validate the functions of LBP and Nrf2 during exposure to ultraviolet radiation.

## Methods

### LBP treatment of HSF cells and UV irradiation

Human skin fibroblast (HSF) cell lines were seeded in 24-well plates maintained in Dulbecco’s modified Eagle’s medium (DMEM) supplemented with 10% fetal bovine serum (FBS; Gibco). Cells were grown in 5% CO_2_ at 37 °C in a humidified incubator for 24 h.

LBP crude extract (purity: 51.87%) was bought from Xi’an Natural Field Bio-Technique Co., Ltd., and PBS was used to prepare 50 g/l LBP crude extract for further use.

To verify the performance of LBP at different concentrations in HSF cells, the cells were treated with 0 (control), 100, 300 and 600 μg/ml LBP. After 24 h, the supernatant was removed, and the cells were washed twice with PBS. The cells were supplemented with DMEM and incubated for 6 h at 37 °C with 5% CO_2_ in a humidified incubator. Plasma and nuclear proteins were purified from the cells using the Qproteme Cell Compartment Kit (QIAGEN). The protein level of Nrf2 was determined via western blotting.

In our previous study [25], concentrations of LBP was < 300 μg/ml did not affect the proliferative activity of HSF cells, but concentrations > 300 μg/ml inhibited cell proliferation. Therefore, to verify the performance of LBP in HSF cells, the cells were treated with 300 μg/ml LBP for different times. Cells were collected, and the protein was purified after incubation for 0.5, 1, 2, 3 and 4 h. The protein levels of p-Nrf2 and GAPDH were determined via western blotting.

The pathway inhibition reagents LY294002 and genistein were used to determine the correlation between LBP performance, p-Nrf2, and various pathways. LY294002 selectively suppresses the PI3K/AKT pathway, inhibiting the generation of p-AKT, while genistein can specifically suppress the process of tyrosine phosphorylation. Cells were incubated in medium alone (control) or with LBP, LBP and genistein, or LBP and LY294002 for 1 or 3 h. After treatment, the supernatant was removed and the protein was purified and measured via western blotting.

For UV irradiation, the primary medium was removed and the cells were washed twice with D-Hanks and then supplemented with an equal volume of PBS to avoid drying. UVA and UVB lamps were used to emit 320–430 nm and 290–320 nm light, respectively. A UVB dosimeter (Sigma High-Tech Co.) was used to monitor the intensity. Based on results from our previous studies [26, 27], the respective dosages of UVA and UVB for this study were 30 J/cm^2^ and 400 mJ/cm^2^. The exposure time was calculated as the UV irradiation dose/UV intensity. After irradiation, the PBS was replaced with fresh medium.

### Determination of the optical absorbance and sun protection factor (SPF)

The LBP was dissolved in 3 ml DMEM at 300 μg/ml. Regular culture medium was used as a control. The optical absorbance of the samples was measured using a luminometer. The optical density was set up from 200 to 400 nm with 5-nm gradient changes. The absorbance at different densities was recorded. SPF for LBP was calculated as previously described [28]. SPF was measured three times, and the mean value was used for the calculation, with the following equation:

$$ SPF\kern0.5em = CF\kern0.5em \times \kern0.5em \sum \limits_{290}^{320} EE\kern0.5em \times \kern0.5em FF\kern0.5em \times \kern0.5em Abs $$

where EE is the erythemal effect spectrum, I is the solar intensity spectrum, Abs is the the absorbance of sunscreen product, and CF is the correction factor (= 10). EE × I is a predetermined constant (Table 1).

**Table 1:** Sun protection factors (SPF). of LBP

Wavelength (nm)	EE × I	Abs	Abs × EE × I
290	0.015	0.192	0.003
295	0.0817	0.177	0.014
300	0.2874	0.169	0.049
305	0.3278	0.151	0.049
310	0.1864	0.131	0.024
315	0.0837	0.117	0.010
320	0.018	0.107	0.002
		Total	0.152
		Total×10	1.520
		SPF for LBP	1.520

### ARE reporter assay

The ARE reporter was designed based on a previous study [29]. The ARE components were integrated into the ML vector to construct the 8ARE-ML reporter vector. The HSF cells were seeded in a 24-well plate and supplemented with DMEM. The 8ARE-ML reporter was transfected into cells using Lipofectamine 2000 (Thermo Fisher) following the manufacturer’s instruction. After incubation for 5 h with the transfection complex, the supernatant was removed and replaced with regular DMEM for 24 h. Genistein and LY-294002 were added to the cells. LBP was also added to the cells. After treatment for 1, 3, 6 and 24 h, the cell culture supernatant was collected and assessed for fluorescent activity using the Secrete-Pair Dual Luminescence Assay Kit.

### Lentivirus preparation

Nrf2 was silenced using the shRNA vector packaged in the lentivirus. Specific oligo sequences were synthesized targeting positions 1176 and 1558 of the Nrf2 gene. The oligo fragments were dissolved in water at 50 μM and paired in a complementary manner by heating for 3 min at 95 °C and cooling at room temperature. The DNA helix was then integrated into the pLVX vector to construct the pLVX-shRNA vector.

293 T cells were seeded in 10-cm plates. When the cells had grown to cover 80–90% of the plate, they were harvested with trypsin and amplified in 15-cm plates. The pLVX-shRNA vector and package vectors (pGag/Pol, pRev and pVSV-G) were transfected into the cells using Lipofectamine 2000 (Thermo Fisher) following the manufacturer’s instructions.

After incubation for 6 h at 37 °C with 5% CO_2_ in a humidified incubator, the supernatant was removed and replaced with regular DMEM. After incubation for 72 h at 37 °C with 5% CO_2_ in a humidified incubator, the cell culture supernatant was collected and centrifuged at 4000 rpm for 4 min. The supernatant was filtered through a 0.45-μm filter and then centrifuged at 20,000 rpm for 2 h. The virus solution was stored at − 80 °C before use. HSF cells were seeded in 96-well plates at 3 × 10^4^ cells/well. The virus solution was diluted in DMEM using a 10× gradient to give 5 different concentrations. The culture medium was removed from each well and supplemented with 100 μl of the virus solutions at different concentrations. Saline was used as a control. Cells were incubated at 37 °C with 5% CO_2_ in a humidified incubator for 24 h. The cell culture supernatant was removed and replaced with 100 μl DMEM. The cells were incubated for 72 h. Flow cytometry was applied to measure the fluorescence, and the virus titer was calculated based on the dilution ratio. RT-PCR and western blotting were performed to detect the level of Nrf2.

### Determination of SOD, ROS, glutathione peroxidase and lipid peroxide levels and cell viability

HSF cells with or without pLVX-shRNA1176 transfection were seeded in 24-well plates and incubated for 24 h at 37 °C with DMEM and 5% CO_2_ in a humidified incubator. After incubation, LBP was added to the cells at 300 μg/ml, followed by further incubation for 24 h. The cells received optical treatment at a dose of 300 mJ/cm^2^ UVB or 25 J/cm^2^ UVA for 24 h.

SOD levels were determined using the SOD Detection Kit (Beyotime). Briefly, 1.5 ml SOD detection buffer was mixed with 800 μl WST-8 and 100 μl enzyme solution to prepare the working solution. Next, 20 μl cell culture supernatant was collected from each sample. The cell culture supernatant was then mixed with 160 μl working solution and 20 μl reaction buffer. The blank1 solution was prepared by mixing 20 μl SOD detection buffer, 160 μl working solution, and 20 μl reaction buffer. The blank2 solution consisted of 40 μl SOD detection buffer. Each mixture was incubated at room temperature for 30 min, and then the OD450 value was detected using a luminometer. The suppression ratio was calculated using the equation:

suppression ratio = (A_blank1_ - A_sample_)/(A_blank1_ - A_blank2_) * 100%

The SOD level was calculated using the equation:

y = 0.0106× + 0.01

where x is 1/SOD level and y is 1/suppression ratio.

To determine the ROS level, the cells were incubated for 30 min with an ROS-specific probe labeled with DHE dye. The cells were washed and resuspended in PBS, and then the fluorescent signal was detected using flow cytometry.

The cells were digested and resuspended in 1 ml PBS. The GSH-PX level was then determined using the Glutathione Peroxidase Detection Kit (Jiancheng) following the manufacturer’s instructions. The OD value at 412 nm was collected using a luminometer, and the level of GSH-PX was calculated.

The LPO level was determined using Nile red staining. The cells were collected and digested with trypsin, then incubated for 5 min with Nile red. The supernatant was removed, and the cells were washed twice with PBS and assessed using a flow cytometer.

The cell viability was determined using the MTT assay. Cells were collected, digested with trypsin, seeded in 96-well plates at 7000 cells/well, and supplemented with DMEM. The cells were incubated for 24 h at 37 °C with 5% CO_2_ in a humidified incubator. MTT was added to the cells for 4 h at 37 °C in the dark. After removal of the supernatant, the formed formazan crystals were dissolved in 100 μl dimethyl sulfoxide, and the absorbance was measured at 490 nm.

### RT-PCR

cDNA was synthesized from total RNA using the M-MLV Reverse Transcriptase Kit (Promega) according to the manufacturer’s instructions. PCR was performed with GoTaq qPCR Master Mix (Promega) with amplification on the ABI 7500 system (Applied Biosystem). GAPDH was used as an internal control.

### Western blotting

Cells were lysed in 1% SDS lysis buffer. The concentration of protein was evaluated using the BCA Protein Assay Kit (Beyotime). The protein was subjected to 10% SDS-PAGE and then transferred onto nitrocellulose membranes. Membranes were blocked with 5% nonfat milk in PBS for 1 h at room temperature and incubated overnight at 4 °C with primary antibody (Abcam). After several washes, the membranes were incubated for 2 h at room temperature in blocking buffer with a secondary antibody coupled to horseradish peroxidase. The complexes were assessed using ECLplus (Amersham Biosciences/GE Healthcare).

### Statistical analysis

For data with a normal distribution, comparisons were performed using independent t-tests, one-way ANOVA and two-way ANOVA. The non-parametric Mann-Whitney U-test, K-S test, Kruskal-Wallis test and Wilcoxon test were performed if the data did not have a normal distribution. Significance was established as *p* < 0.05. SPSS and GraphPad Prism software was used for the statistical analysis.

## Results

### LBP functions by regulating Nrf2

**Fig. 1:**
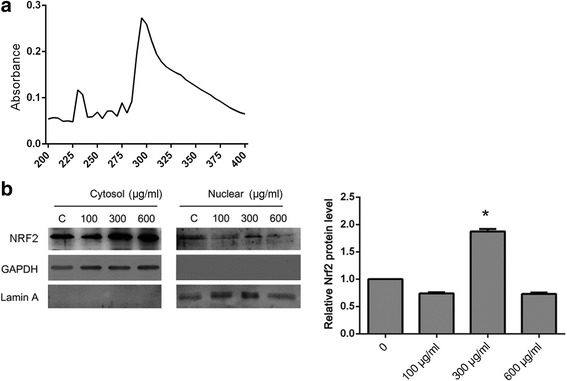
Relevant characteristics of LBP and Nrf2. **a** – The optical absorbance of LBP at different optical densities. **b** – The protein levels Nrf2 in the cytosol and the nucleus. The plotted graph indicates the level of nuclear Nrf2 at different concentrations of LBP. Experiments were repeated at least in triplicates, *p < 0.05

LBP was first detected based on the differences in the optical absorbance signal at different densities (Fig. 1a). The absorbance of the LBP solution peaked at ~ 300 nm, with the absorbance values are all under 0.2. The SPF of LBP (Table 1) was 1.520.

Since ultraviolet light ranges in wavelength from 200 to 400 nm, the capability of LBP to protect cells from ultraviolet radiation damage does not rely on physical depletion of radiation. Therefore, it should have a regulatory activity in cells. Nrf2 was targeted as a molecule that might be important for this because it is significantly expressed in the cytoplasm but also in the nucleus (Fig. 1b). The level of Nrf2 was not significantly different between the different treatments. However, the level of nuclear Nrf2 was significantly differentially expressed in response to treatment with 300 μg/ml LBP. Since Nrf2 normally performs its functions when it translocates to the nucleus, it can be suspected that LBP regulates Nrf2 translocation and this has a function in protection against ultraviolet light.

### P-Nrf2 is activated by LBP and its functions correlate with nuclear translocation

**Fig. 2:**
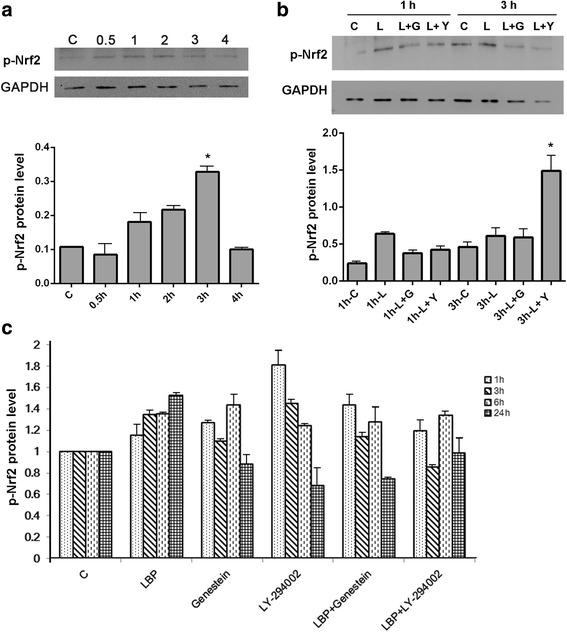
Response of p-Nrf2 to treatment with LBP and inhibitors. **a** – The protein level of p-Nrf2 in HSF cells treated with LBP for 0 (control, C), 0.5, 1, 2, 3 or 4 h. **b** – The protein level of p-Nrf2 in HSF cells in medium (1 h-C, 3 h-C, control) or treated with LBP alone for 1 h or 3 h (1 h-L, 3 h-L), LBP plus genistein for 1 h or 3 h (1 h-L + G, 3 h-L + G), or LBP plus LY294002 for 1 h or 3 h (1 h-L + Y, 3 h-L + Y). The protein level of p-Nrf2 was determined via western blotting. **c** – The nuclear level of p-Nrf2 determined using the ARE reporter for 1, 3, 6 or 24 h, as shown.. Experiments were repeated at least in triplicate, *p < 0.05

Since Nrf2 normally performs its functions when it is phosphorylated, we conducted western blotting to determine the correlation between p-Nrf2 and LBP levels. The results showed that the p-Nrf2 level was elevated after LBP treatment for 1 h, but that it had decreased to normal levels after 4 h (Fig. 2a). The p-Nrf2 level reached its highest level after 3 h. This was almost 3-fold higher than in the control group. Therefore, treatment with 300 μg/ml LBP for 3 h was optimal to activate Nrf2, which is consistent with the results of our previous study [30].

The phosphorylation process was further studied using the inhibition reagents LY294002 and genistein. The cells were incubated with medium alone (control), or with LBP, LBP and genistein, or LBP and LY294002 for 1 or 3 h (Fig. 2b). Results are described relative to the control for 1 or 3 h. After 1 or 3 h, LBP treatment alone had caused a slight increase in p-Nrf2 level. Treatment with LBP and genistein for 1 h yielded higher levels than the control, but lower levels than with LBP alone. After 3 h treatment, this difference was less. Treatment with LBP and LY294002 for 1 h had a similar effect, but treatment with this combination for 3 h let to a significant (3-fold) increase in p-Nrf2 levels.

Genistein blocks tyrosine phosphorylation of Nrf2 and LY294002 targets the PI3K/AKT pathway used for nuclear translocation. The reduced p-Nrf2 level in response to genistein and LBP treatment suggests that Nrf2 phosphorylation might occur via tyrosine phosphorylation, and this should correlate to reduced activity. The increased level in response to LY294002 and LBP treatment demanded more investigation: by inhibiting the PI3K/AKT pathway, LY294002 probably inhibits the nuclear translocation of p-Nrf2, i.e., it affects its capability to be active in the nucleus but not its level.

Therefore, we used the specifically designed ARE reporter to determine the activity of nuclear p-Nrf2 (Fig. 2c). The signal gradually increased showing that LBP treatment clearly increased the activity of p-Nrf2. After 24 h of either genistein or LY294002 treatments, the activity of the nuclear p-Nrf2 had decreased compared with the control group. Moreover, the reduced activity could not be reversed by LBP.

These results confirm that LBP could enhance the phosphorylation of Nrf2 to increase nuclear p-Nrf2 levels and, in the absence of inhibitors, thus improve its performance. As noted, genistein blocks tyrosine phosphorylation of Nrf2, leading to a reduced level and activity of p-Nrf2. LY294002 targets the PI3K/AKT pathway, resulting in higher cytoplasmic levels of p-Nrf2, but probably also suppressing the nuclear translocation of p-Nrf2, which we believe leads to reduced activity. This indicates that the functions of Nrf2 rely on both phosphorylation and nuclear translocation.

### LBP depends on Nrf2 to regulate physiological features

**Fig. 3:**
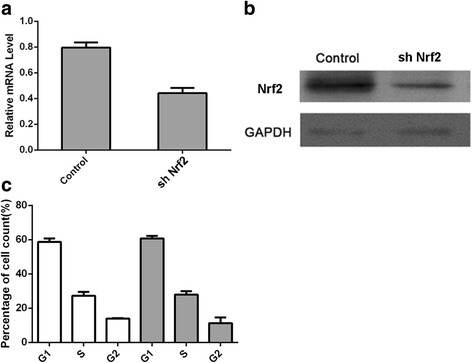
Performance of gene silencing of Nrf2. The protein level of Nrf2 was specifically decreased, but the cell cycle remained unchanged. Experiments were repeated at least in triplicate

To investigate the functions of Nrf2 during ultraviolet damage, Nrf2 was silenced by transfecting HSF cells with shRNA. Positions 1176 and 1558 were both found to inhibit its expression. We chose a position 1176-specific shRNA for further studies because of its obvious ability to silence Nrf2 expression (Figs. 3a and b). The sh-Nrf2 decreased the expression of Nrf2 to 50% of that in the control group. However, the expression level of Nrf2 did not correlate with the cell cycle. Hardly any difference in cell counts in the different phases was observed between the two groups.

**Fig. 4:**
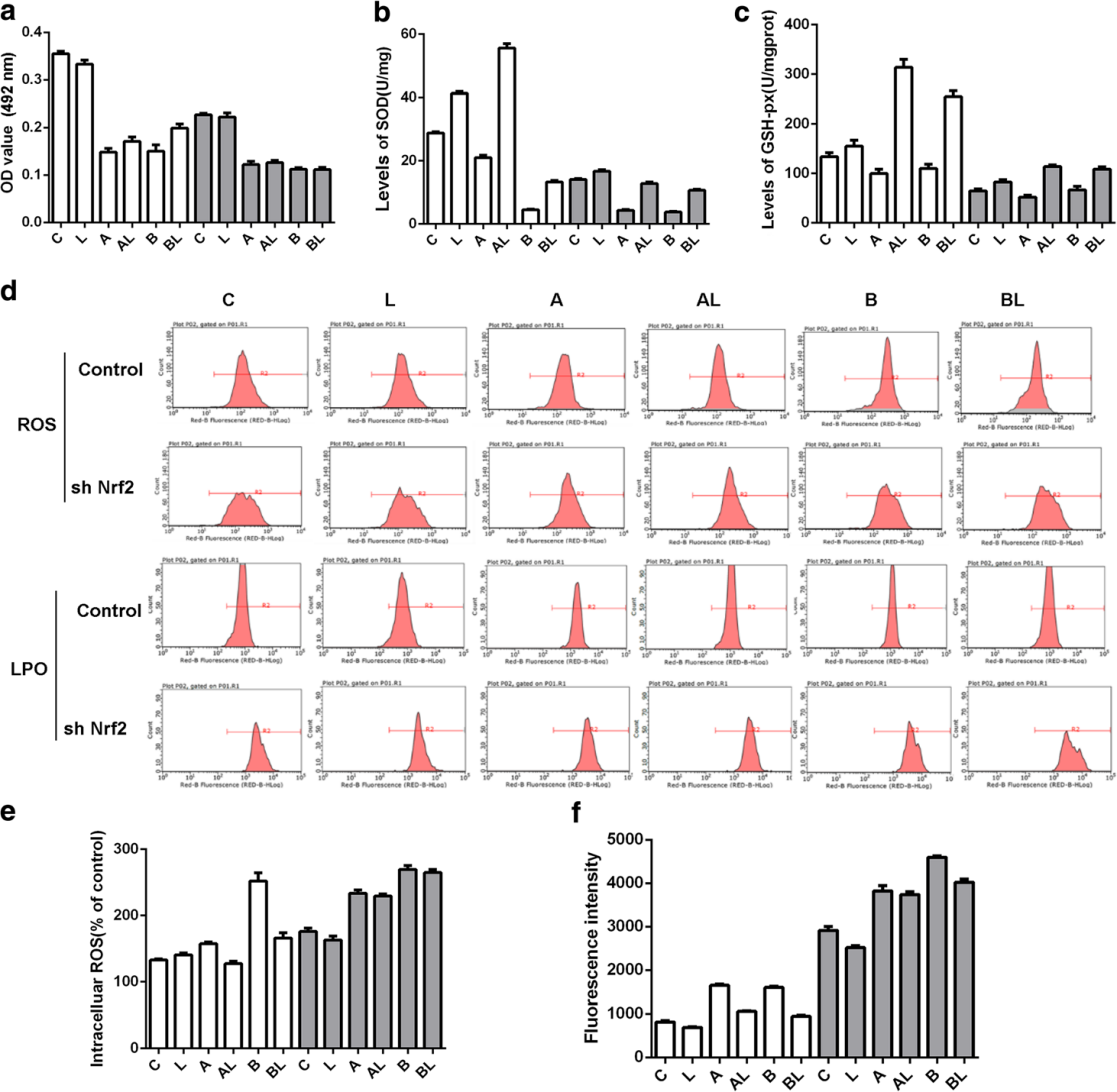
Levels of important physiological factors and cell viability in response to UV radiation. **a** – Cell viability determined using the MTT assay. **b** – The levels of SOD. **c** – The levels of GSH-PX. D and **e** – The levels of ROS. **d** and **f** – The levels of lipid peroxide. Experiments were repeated at least in triplicate. For all: White plots refer to cells without Nrf2 silencing; grey plots refer to cells with NRF2 silencing. **c** – control group; **a** – UV at a dose of 25 J/cm^2^; **b** – UV at a dose of 300 mJ/cm^2^; L – LBP; AL – LBP with UV at a dose of 25 J/cm^2^; BL – LBP with UV at a dose of 300 mJ/cm^2^

Cell viability was continually determined using the MTT assay (Fig. 3c). Radiation-induced damage reduced cell viability, while LBP slightly but non-significantly enhanced cell viability. Silencing Nrf2 decreased cell viability, and this decrease could not be reversed by LBP (Fig. 4a).

Similarly, the levels of SOD and GSH-px obviously decreased after the radiation treatment (Fig. 4b and C). With increasing intensity of radiation, the level of SOD decreased more dramatically. Silencing Nrf2 led to reduced level of both SOD and GSH-px. LBP could increase the level of both SOD and GSH-px, irrespective of whether Nrf2 was silenced.

Conversely, the ROS level increased after radiation treatment, and it positively correlated with the intensity of the radiation (Fig. 4e). LBP functioned to decrease ROS levels. When Nrf2 was silenced, it induced slight increases in the ROS level in the control and LBP treatment group. However, the ROS level increased significantly after radiation treatment, and this effect could not be reversed by LBP. Radiation led to higher LPO levels, which could be prevented by LBP (Fig. 4f). However, the LPO level increased significantly when Nrf2 was silenced. Under this condition, the LPO level positively correlated with the intensity of radiation, and LBP only marginally lowered the LPO level.

Based on these results, Nrf2 should be important for the regulation of the mentioned physiological features. Although LBP could help to improve the situation, its functions were sometimes lost when Nrf2 was silenced. Therefore, the functions of LBP rely on Nrf2 to some extent.

## Discussion

In this study, we conducted assays using HSF cells to identify the functions of LBP and Nrf2 in the protective effect against UVR-induced damage. The results confirmed that Nrf2 should be phosphorylated to p-Nrf2 by tyrosine phosphorylate before it performs its functions, and that LBP could enhance the expression level of nuclear p-Nrf2. The nuclear translocation process might be related to the PI3K/AKT pathway. When Nrf2 was silenced, LBP could only partially improve the situation caused by UVR. Therefore, we propose that LBP can protect cells from UVR, and that Nrf2 is an important regulator of this protective effect.

LBP has been used as a medicine for centuries in China, and it is presumed to have low toxicity [31]. However, the effective components of LBP have not been clearly characterized. We used LBP at 300 μg/ml. A lower dose of LBP might be not sufficient to activate Nrf2, but higher doses might lead to cell toxicity and preclude any protective effect.

A previous study focused on the active components of LBP during the development of non-alcoholic steatohepatitis. The results showed that although L-arabinose and β-carotene, the active components of LBP, exhibited a partially hepatoprotective effect, LBP provided more powerful protection in a steatosis model [32]. Thus, the stronger protective effect of LBP might be ascribed to the synergistic effect of the different composition. Clarification of the key active components in LBP and possible synergistic mechanisms merits further study to avoid any side effects and efficiently promote its functions.

LBP has also been reported to exhibit antioxidant activity and to protect multiple tissues against various toxicities through inhibition of oxidative stress [33, 34]. We confirmed that LBP can effectively protect against UVB-induced keratinocyte damage in immortalized human keratinocytes (HaCaT cells) [31]. In this study, the protective effects of LBP and the underlying mechanisms against UVR-induced damage were investigated in HSF cells, and we obtained results consistent with those of the previous study [31].

UVR consisting of UVA and UVB caused significant decreases in cell viability due to excessive intracellular ROS production. The Nrf2/ARE pathway is a primary cellular defense controlling the oxidative stress response, which regulates numerous antioxidant-related genes. Therefore, Nrf2 should be an important regulator of protection against UV damage in the epidermis [35].

Our previous study showed that the protective effects of LBP are partly due to activation of the Nrf2/ARE pathway and partly due to inhibition of UVB-induced p38 MAP pathway activation [31]. The regulatory effect of LBP on Nrf2 was recently indicated to include the prevention of high fat-induced insulin resistance through upregulation of the PI3K/AKT/Nrf2 signaling pathway in mice [21].

In our study, the inhibition reagents LY294002 and genistein were used to explore the activation of Nrf2. Genistein inhibits tyrosine phosphorylation, and thereby decreases the levels of p-NRF2, supressing the Nrf2/ARE pathway. LY294002 is a commonly used inhibitor of PI3K/AKT. Using LY294002 resulted in higher overall p-Nrf2 levels, but the level of nuclear p-Nrf2 gradually decreased, indicating that PI3K/AKT might be an important pathway during the phosphorylation and translocation of Nrf2.

Note that our results were slightly different from those of Yang et al. [10], who showed that LBP provided no clear time-correlated improvement in p-Nrf2. Thus, the level of p-Nrf2 was irregular during simultaneous treatment with LBP and the inhibitor. Therefore, the regulation of LBP might not only depend on the PI3K/AKT pathway, but also other pathways or cytokines that are involved in the process.

LBP significantly decreased ROS levels and correspondingly increased cell viability. However, when Nrf2 was silenced, the protective ability of LBP was ineffective, which indicated that the functions of LBP in lowering ROS levels were mainly mediated by Nrf2.

By contrast, LBP also protected cells from UV damage by enhancing the levels of SOD and GSH-PX. Our results are consistent with those from a previous study [1]. SOD and GSH-PX are important antioxidants, but they are also important for protecting the skin from photo-damage. Since LBP enhanced their levels, the level of LPO, which is indicative of UV damage, was correspondingly reduced. However, in contrast to the results for ROS and cell viability, although silencing Nrf2 reduced SOD and GSH-PX levels, LBP increased them regardless of whether NRF2 was silenced. These results suggested that the ability of LBP to enhance the activities of SOD and GSH-PX did not simply rely on Nrf2. Other cytokines might also be involved in the process and be regulated by LBP. However, after silencing of Nrf2, the levels of SOD and GSH-PX significantly decreased, and the level of LPO significantly increased. Nrf2 can thus be proposed as a very important factor for regulating the levels of SOD, GSH-PX and LPO.

## Conclusions

Our results confirm that LBP performs its functions by regulating Nrf2, and that the functions of Nrf2 rely on both phosphorylation and nuclear translocation. However, the functions of LBP also rely on Nrf2 to some extent. This study confirmed the importance of LBP in regulating Nrf2 to protect HSF cells from UV damage. Our results also suggest that LBP may have other targets besides Nrf2. Investigations of such candidate targets will further elucidate the mechanism of LBP’s protective effect against UV damage. Because LBP contains 6 kinds of monosaccharides, a clarification of key active components and potential synergistic mechanisms should also be addressed in future studies.

## Abbreviations

ARE: Antioxidant response element
DMEM: Dulbecco’s modified Eagle’s medium
GSH-PX: Glutathione peroxidase
LBP: *Lycium barbarum* polysaccharide
LPO: Lipid peroxide
Nrf2: Nuclear factor erythroid 2-related factor 2
ROS: Reactive oxygen species
SOD: Superoxide dismutase
SPF: Sun protection factor
UVR: Ultraviolet radiation
